# Decreasing Alexithymia Severity & Improving Emotion Regulation in Ukrainian Military Cadets

**DOI:** 10.1192/j.eurpsy.2026.10787

**Published:** 2026-07-31

**Authors:** A. Haydabrus, S. Larionov

**Affiliations:** 1 V. N. Karazin Kharkiv National University, Kharkiv, Ukraine; 2 Universitat de Autonoma de Barcelona, Barcelona, Spain; 3 Kharkiv National University of Internal Affairs, Kharkiv, Ukraine

## Abstract

**Introduction:**

Alexithymia (A), a difficulty in identifying and describing emotions, affects about 10% of the population and up to 40% of individuals with PTSD (Preece et al., 2022; Putica, 2024). Military personnel are particularly vulnerable, with A strongly assotiated with psychological distress and PTSD. Interventions such as cognitive reappraisal and affect labeling may reduce avoidance behaviours and improve emotion regulation, anxiety, and depression (Torunsky et al., 2023; Scarpazza et al., 2022; Preece et al., 2023; Meuleman et al., 2023).

**Objectives:**

To evaluate the effects of a mobile application practicing cognitive reappraisal and affect labeling on alexithymia, emotion regulation, and mental health outcomes in cadets.

**Methods:**

Twenty-four Ukrainian military cadets (mean age 20.7 years, range 17–30) completed baseline and one-month follow-up surveys. Both surveys included the Toronto Alexithymia Scale–Difficulty Identifying Feelings, Difficulties in Emotion Regulation Scale-16, Generalized Anxiety Disorder-7, Patient Health Questionnaire-9, Connor–Davidson Resilience Scale-10, and PTSD Checklist-5.

**Results:**

Seventeen participants completed the study (7 males, 41.2%; 10 females, 58.8%). At baseline, 47% were alexithymic. 47% of them had moderate–severe anxiety, and 29% had moderately severe depression, exceeding general population rates.

After one month (Figure 1), 65% showed reduced alexithymia (1A), 53% reduced or stable emotion regulation (1B), and 65% reduced or stable PTSD severity (1F). Anxiety (1C) and depression (1D) worsened in 53% and 59%, respectively, likely reflecting job-related stress. Resilience declined in 53% (1E), warranting further analysis of occupational factors and potential data anomalies.

Figure 2 shows that participants with reduced A (2A) had a mean TAS-DIF decrease of 3.36±2.77, those with improved ER (2B) a decrease of 7.42±5.28, and those with reduced PTSD severity (2F) a decrease of 2.57±2.06.

**Image 1:**
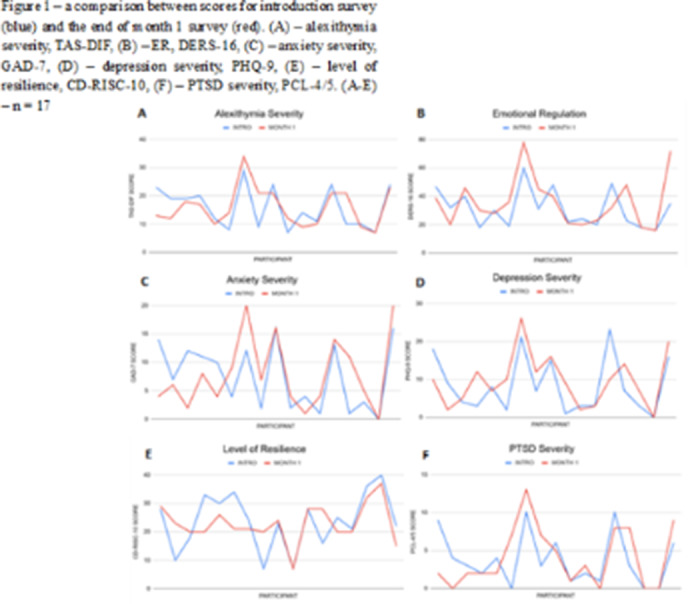
Image 1: Long description.

**Image 2:**
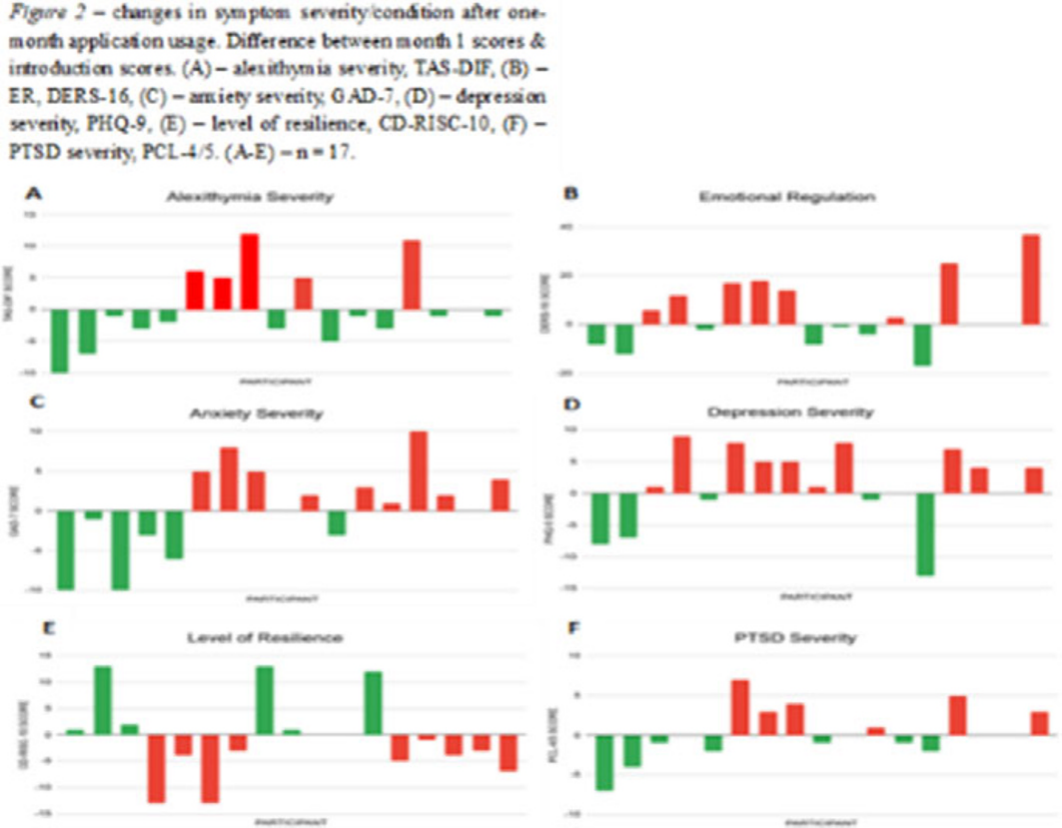
Image 2: Long description.

**Conclusions:**

This study demonstrates associations between A and broader mental health difficulties. Enhancing connectivity between limbic and prefrontal regions through reappraisal and affect labelling may reduce alexithymia severity and improve ER.

**Disclosure of Interest:**

None Declared

## Long descriptions

### Image 1: Long description

Six line graphs arranged in a two-column by three-row grid. Each graph plots participant scores on the y-axis against individual participants on the x-axis. A blue line represents the I N T R O survey and a red line represents the M O N T H 1 survey.

* Panel A: Alexithymia Severity (T A S - D I F score). The y-axis ranges from 0 to 40. Both lines show high variability with a significant peak around the middle participants.

* Panel B: Emotional Regulation (D E R S - 1 6 score). The y-axis ranges from 0 to 80. The red line shows a higher peak than the blue line in the center of the data set.

* Panel C: Anxiety Severity (G A D - 7 score). The y-axis ranges from 0 to 20. The red line generally trends higher than the blue line for the latter half of the participants.

* Panel D: Depression Severity (P H Q - 9 score). The y-axis ranges from 0 to 30. Both lines fluctuate significantly, with the red line showing a sharp peak in the first third of the group.

* Panel E: Level of Resilience (C D - R I S C - 1 0 score). The y-axis ranges from 0 to 40. The blue line shows more extreme troughs than the red line.

* Panel F: P T S D Severity (P C L - 4 / 5 score). The y-axis ranges from 0 to 15. The red line shows a prominent peak where the blue line remains relatively low.

Navigate back to Image 1:.

### Image 2: Long description

The figure consists of six bar charts labeled A through F. Each chart plots the difference between month 1 scores and introduction scores for individual participants. Positive changes are shown in red and negative changes in green, except for panel E where the color logic for resilience is inverted.

* Panel A: Alexithymia Severity (T A S - D I F score). Shows a mix of decreases and increases, with the largest increase reaching approximately 12 points.

* Panel B: Emotional Regulation (D E R S - 16 score). Displays mostly positive changes (increases in score), with several participants showing increases between 15 and 40 points.

* Panel C: Anxiety Severity (G A D - 7 score). Shows varied results with several participants decreasing by 10 points and others increasing by up to 10 points.

* Panel D: Depression Severity (P H Q - 9 score). Shows a majority of participants with increased scores, peaking around 9 points, while a few show significant decreases down to negative 13.

* Panel E: Level of Resilience (C D - R I S C - 10 score). Features several participants with increased resilience (green bars up to 13 points) and several with decreased resilience (red bars down to negative 13 points).

* Panel F: P T S D Severity (P C L - 4 / 5 score). Shows mostly small fluctuations, with one participant increasing by 7 points and another decreasing by 7 points.

Navigate back to Image 2:.

